# The Multifaceted Roles of Proline in Cell Behavior

**DOI:** 10.3389/fcell.2021.728576

**Published:** 2021-08-12

**Authors:** Eduardo J. Patriarca, Federica Cermola, Cristina D’Aniello, Annalisa Fico, Ombretta Guardiola, Dario De Cesare, Gabriella Minchiotti

**Affiliations:** Stem Cell Fate Laboratory, Institute of Genetics and Biophysics “A. Buzzati Traverso”, Consiglio Nazionale delle Ricerche, Naples, Italy

**Keywords:** proline metabolism, cell plasticity, extracellular proteins, energy source, antistress activity, neural toxicity, signaling modulators, metabolism

## Abstract

Herein, we review the multifaceted roles of proline in cell biology. This peculiar cyclic imino acid is: ***(i)*** A main precursor of extracellular collagens (the most abundant human proteins), antimicrobial peptides (involved in innate immunity), salivary proteins (astringency, teeth health) and cornifins (skin permeability); ***(ii)*** an energy source for pathogenic bacteria, protozoan parasites, and metastatic cancer cells, which engage in extracellular-protein degradation to invade their host; ***(iii)*** an antistress molecule (an osmolyte and chemical chaperone) helpful against various potential harms (UV radiation, drought/salinity, heavy metals, reactive oxygen species); ***(iv)*** a neural metabotoxin associated with schizophrenia; ***(v)*** a modulator of cell signaling pathways such as the amino acid stress response and extracellular signal-related kinase pathway; ***(vi)*** an epigenetic modifier able to promote DNA and histone hypermethylation; ***(vii)*** an inducer of proliferation of stem and tumor cells; and ***(viii)*** a modulator of cell morphology and migration/invasiveness. We highlight how proline metabolism impacts beneficial tissue regeneration, but also contributes to the progression of devastating pathologies such as fibrosis and metastatic cancer.

## Introduction

**FIGURE 1:**
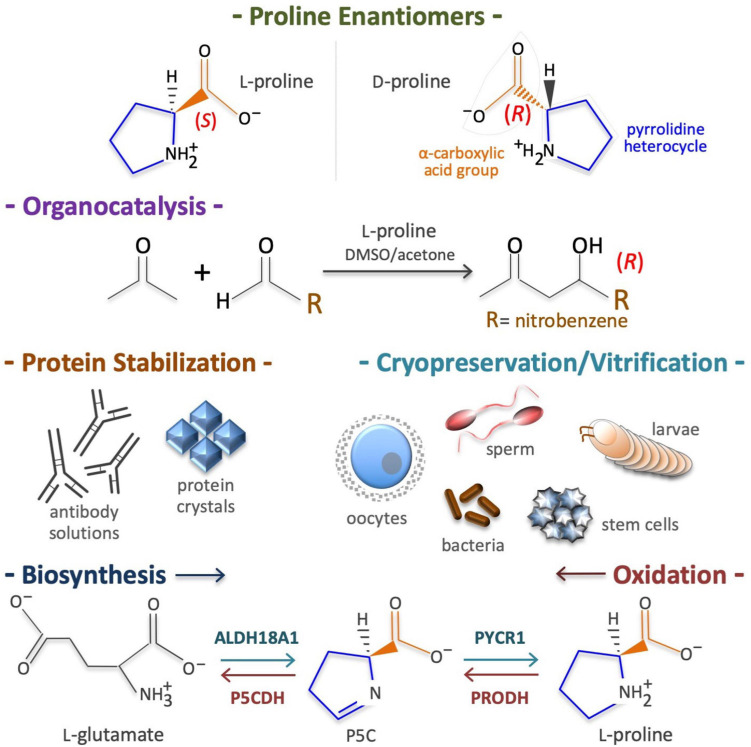
Proline structure, uses, biosynthesis and degradation/oxidation. Chirality of pyrrolidine-2-carboxylic acid **(*top*)**, known as proline (CAS: 147-85-3, EC: 205-702-2, CHEBI: 17203, HMDB0000162, MW = 115.13 g/mol). Of the two enantiomers (L and D) living cells metabolize predominantly the L-proline enantiomer **(*top*)**. Proline is an organocatalyst used to synthesize enantiopure drugs **(*middle top*)**. Proline is also a potent chemical chaperone able to stabilize proteins in their natural conformation and thus, it is used to cryopreserve living cells/organisms **(*middle bottom*)**. Due to its pyrrolidine ring structure, the enzymes involved in *de novo* L-proline biosynthesis, namely aldehyde dehydrogenase 18 family member A1 (ALDH18A1) and pyrroline-5-carboxylate reductase 1 (PYCR1), as well as the enzymes involved in L-proline oxidation, namely proline dehydrogenase (PRODH) and the pyrroline-5-carboxylate dehydrogenase (P5CDH), are highly specific **(*bottom*)**.

In 1900, Richard M. Willstätter reported the synthesis of (*S*)-pyrrolidine-2-carboxylic acid, better known as L-Pro. Town (1928) reported the purification of L-Pro from gliadin proteins, and Levine (1959) reported that nitrous acid destroys all amino acids apart from L-Pro in hydrolyzed gelatins, and highlighted its unusual structure. L-Pro is a small (115.13 g/mol), cyclic, non-polar, non-toxic, odorless, sweet-tasting imino acid, with unique physicochemical proprieties and numerous biotechnological applications (Figure 1). For instance, acting as an enantioselective organocatalyst, L-Pro makes possible the synthesis of therapeutically active enantiopure drugs (Table 1). Moreover, acting as a chemical chaperone, L-Pro can prevent protein aggregation/fibrillation, and is therefore used to stabilize monoclonal antibodies, to generate protein crystals (Table 1), and for the cryopreservation of biological specimens, including stem cells and oocytes (Table 1). Due to its peculiar cyclic structure, its metabolism relies on specific enzymes. For instance, in mammalian cells L-Pro is synthesized from L-glutamate in a two-step intramitochondrial process catalyzed by aldehyde dehydrogenase 18 family member A1 (ALDH18A1) and pyrroline-5-carboxylate reductase 1 (PYCR1) enzymes (Figure 1), whereas it is oxidized to L-glutamate in a two-step intramitochondrial process catalyzed by proline dehydrogenase (PRODH) and pyrroline-5-carboxylate dehydrogenase (P5CDH) enzymes (Figure 1).

**TABLE 1:** Proline in drug synthesis, protein stabilization and cryopreservation.

Enantioselective organocatalysis^1^		
Compound synthesized	Type of chemical reaction	References
Prostaglandin PGF2alpha	Corey’s synthesis	Coulthard et al. (2012)
Pyrans, and thiopyrans	Methylene ketones and α,β-unsaturated nitriles	Elnagdi and Al-Hokbany (2012)
(R)-4-(4-Methoxy-phenylamino)-6-methyl-heptan-2-one	Ketones, aldehydes and Meldrum’s acid	List and Castello (2001)
Oxazolidinones	Asymmetric aldol reaction	List et al. (2004)
Delta(1)(2)-prostaglandin J(3)	–	Pelss et al. (2018)
–	Hajos–Parrish–Eder–Sauer–Wiechert (HPESW) reaction	Rance and Khlobystov (2014)
Erythromcin 1	Stereospecific aldolization	Agami et al. (1987)
–	Michael addition of malonate anions to enones and enals	Yamaguchi et al. (1996)
**Protein stabilization^2^**		
**Biological sample**	**Stabilizing medium**	
Insulin	L-Proline (0.05–0.25 M)	Choudhary et al. (2015)
Insulin and lysozyme	L-Proline/sorbitol	Choudhary et al. (2016)
Antibodies solutions (mAb)	L-Proline (up to 1.3 M)	Hung et al. (2018)
Huntingtin (polyQ tracts)	L-Proline	Ignatova and Gierasch (2006)
Lysozyme, xylose isomerase, P5CDH	L-Proline (2–3 M) and protein-crystallization solution	Pemberton et al. (2012)
Lysozyme	L-Proline (1.5–4.0 M)	Samuel et al. (1997), Samuel et al. (2000)
Lactate dehydrogenase	L-Proline (up to 4.0 M)	Wang and Bolen (1996)
**Cryopreservation/vitrification^3^**		
**Specimen**	**Freezing medium**	
Human mesenchymal stem cells (hMSCs)	L-Proline, methylcellulose, ectoin	Freimark et al. (2011)
Human endothelial cells	L-Proline	Sun et al. (2012)
Ram sperm	L-Proline	Sanchez-Partida et al. (1998)
Mammalian oocytes	L-Proline/ethylene glycol/DMSO	Zhang et al. (2016a, b)
Donkey semen	L-Proline	Li et al. (2021)
Mice oocytes	L-Proline oligomers (L-Pro_*n*_)	Qin et al. (2020), Treleaven et al. (2021)
Lactic acid bacteria	L-Proline/glycerol	Qiao et al. (2018)
Vesicles of sarcoplasmic reticulum from lobster muscle	L-Proline (more effective than glycerol or DMSO)	Rudolph and Crowe (1985)

*Applications in: ^1^pharmaceutical industry, ^2^pharmacological therapy, and ^3^biomedical research, regenerative medicine.*

## Proline in Extracellular Matrix Production

L-Proline residues constitute nearly 6% of the human proteome, mainly concentrated in L-Pro-rich proteins, with up to 1 × 10^4^ L-Pro-rich motifs/stretches occurring in 1.8 × 10^4^ human proteins (Morgan and Rubenstein, 2013; Mandal et al., 2014). In addition to a high L-Pro content (up to 50% of total residues), L-Pro-rich peptides/proteins share extracellular localization (secreted proteins), a dedicated translation factor (EIF5A), and a requirement for timely L-Pro-tRNA loading (Doerfel et al., 2013; Gutierrez et al., 2013; Wu et al., 2020; Faundes et al., 2021). Free L-Pro is derived from dietary sources (animal collagens or vegetable extensins) or from *de novo* biosynthesis (Figure 1), which relies on mitochondrial generation of reduced nicotinamide adenine dinucleotide phosphate (NADPH) (Tran et al., 2021; Zhu et al., 2021). Why so many extracellular proteins are rich in L-Pro is a fascinating question; L-Pro residues destabilize α-helices and β-sheets protein secondary structures, enables turns and poly-Pro helices, and are major ‘disorder-promoting’ residues in intrinsically disordered proteins (Theillet et al., 2013; Alderson et al., 2018; Mateos et al., 2020).

### Matrix Collagens

Collagens constitute ∼30% of total human proteins (Smith and Rennie, 2007), and are secreted by cells of CTs such as bone, cartilage, tendon, ligament, and interconnected fluid-filled CTs (Benias et al., 2018) that support and connects all other tissues (epithelial, muscular, etc.). Collagen synthesis is highly dependent on L-Pro availability (∼170 μM in plasma) (Psychogios et al., 2011), and inherited mutations in *ALDH18A1* or *PYCR1* (*de novo* L-Pro biosynthesis) are a cause of abnormal CT development (Table 2). Extrinsic (dietary) L-Pro is essential during adult life to preserve bone density in a mice model of osteoporosis (Nam et al., 2016), collagen deposition in rats, pigs, chickens and fish (Li and Wu, 2018; He et al., 2021), and L-Pro homeostasis in humans (Jaksic et al., 1990; Bertolo and Burrin, 2008).

**TABLE 2:** Diseases associated with defects in genes involved in the proline metabolism.

Process	Gene	Syndrome	*	OMIM	Phenotype	References
Proline biosynthesis	*ALDH18A1* (*P5CS*)	Cutis laxa 3	AD	616603	Wrinkled and thin skin, cataracts, joint hyperlaxity	Jukkola et al. (1998), Fischer-Zirnsak et al. (2015), Bhola et al. (2017)
		Cutis laxa type IIIA	AR	219150	Growth retardation, poor postnatal growth,	de Barsy et al. (1968), Baumgartner et al. (2000), Bicknell et al. (2008), Skidmore et al. (2011), Fischer et al. (2014)
		Spastic paraplegia 9A	AD	601162	Short stature, skeletal abnormalities, cataracts	Slavotinek et al. (1996), Seri et al. (1999), Coutelier et al. (2015), Panza et al. (2016)
		Spastic paraplegia 9B	AR	616586	Short stature, delayed psychomotor development	Coutelier et al. (2015), Magini et al. (2019)
	*PYCR1* (*P5CR1*)	Cutis laxa, type IIB	AR	612940	Aged appearance, joint hyperextensibility, osteopenia	Guernsey et al. (2009), Reversade et al. (2009), Kretz et al. (2011)
		Cutis laxa type IIIB	AR	614438	Growth retardation, cutis laxa, aged appearance	Reversade et al. (2009), Lin et al. (2011)
	*PYCR2* (*P5CR2*)	Leukodystrophy, hypomyelinating, 10	AR	616420	Poor overall growth, malformed ears, cerebral atrophy	Nakayama et al. (2015), Zaki et al. (2016), Patel et al. (2021)
Proline degradation	*PRODH*	Hyperprolinemia, type I	AR	239500	Neurologic defects, mental retardation, schizophrenia	Campbell et al. (1997), Jacquet et al. (2002, 2003, 2005)
		Schizophrenia susceptibility 4	AD	600850	Psychosis, hallucinations, delusions, erratic behavior	Karayiorgou et al. (1995), Yoon et al. (2016)
	*P5CDH (ALDH4A1)*	Hyperprolinemia, type II	AR	239510	Recurrent seizures, mental retardation, epilepsy	Valle et al. (1974, 1976), Geraghty et al. (1998), Kaur et al. (2021)
Proline transport	*SLC6A20*	Hyperglycinuria	AD	138500	Renal oxalate stones, renal colic	Scriver (1968), Greene et al. (1973), Broer et al. (2008)
		Iminoglycinuria, digenic	AR, DR	242600	Hyperprolinuria, hyperhydroxyprolinuria	Tancredi et al. (1970)
	*SLC6A19 (B°AT1)*	Hartnup disorder	AR	234500	Short stature, intermittent cerebellar ataxia, psychosis	Kleta et al. (2004), Seow et al. (2004)
		Hyperglycinuria	AD	138500	Renal oxalate stones, renal colic	Scriver (1968), Greene et al. (1973), Broer et al. (2008)
		Iminoglycinuria, digenic	AR, DR	242600	Hyperprolinuria, hyperhydroxyprolinuria	Tancredi et al. (1970)
	*SLC36A2 (PAT2)*	Hyperglycinuria	AD	138500	Renal oxalate stones, renal colic	Scriver (1968), Greene et al. (1973), Broer et al. (2008)
		Iminoglycinuria, digenic	AR, DR	242600	Hyperprolinuria, hyperhydroxyprolinuria	Tancredi et al. (1970)
	*SLC6A7 (PROT)*	Unknown		606205	Unknown	Fremeau et al. (1992), Shafqat et al. (1995), Velaz-Faircloth et al. (1995)

*Inheritance.

*AD, autosomal dominant; AR, autosomal recessive; DR, digenic recessive.*

### Antimicrobial Peptides

**FIGURE 2:**
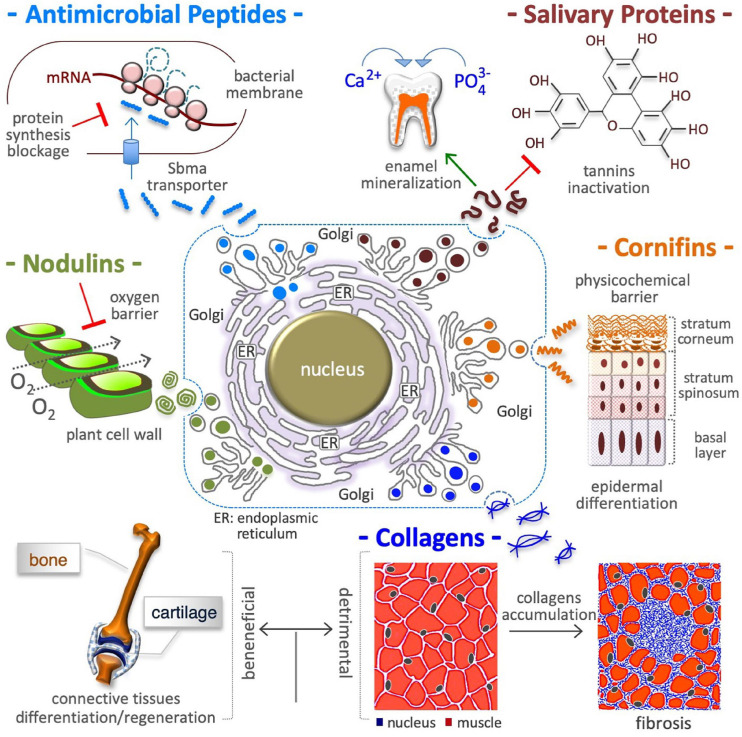
Proline in extracellular matrix production. Proline is a crucial building block of antimicrobial peptides, salivary proteins, epidermal cornifins, interstitial collagens, and plant nodulins. These proteins are all rich in proline residues (with up to 50% of total amino acids) and are all secreted in the extracellular space. In addition to shape cell/tissue microenvironment/architecture (fibrillar collagens), proline-rich proteins contribute to innate immunity (antibiotic activity) by inhibiting bacterial protein synthesis **(*top left*)**, to diet selection (astringency) by binding polyphenolic tannis **(*top right*)** and, to teeth health by inducing enamel mineralization and preventing bacterial attacks **(*top right*)**, to selective permeation (barrier of water, O_2_) by nodulins in N_2_ fixing root nodules of leguminous plants **(*middle left*)**, and by cornifins in skin **(*middle right*)**, and to signaling mechanical forces (ECM stiffness). The accumulation of interstitial collagens leads to pathological fibrosis and occurs in different tumoral tissues **(*bottom right*)**.

L-Proline-rich antimicrobial peptides (PrAMPs), involved in innate immunity, are the first line of defense against infections (Graf and Wilson, 2019), and they contain up to 50% L-Pro residues, and are secreted by insects, crustaceans and mammals (Table 3; Mishra et al., 2018). Mechanistically, PrAMPs are channeled by the peptide antibiotic transporter SbmA into the bacterial cytoplasm (Mattiuzzo et al., 2007; Runti et al., 2013), where they bind ribosomal proteins and inhibit protein synthesis (Figure 2 and Table 3; Graf et al., 2017; Graf and Wilson, 2019; Baliga et al., 2021).

**TABLE 3:** Proline-rich antimicrobial peptides.

Peptide	Isolated from	Susceptible organism	Molecular mechanism	References
Apidaecins (18–20 aa)	*Apis mellifera* lymph fluid	Antibacterial (Gram-) Human and plant pathogens	Protein translation inhibition -Trapping RF1 and RF2 -Blocks assembly of 50S	Casteels et al. (1989), Li et al. (2006), Krizsan et al. (2015), Chen et al. (2017), Florin et al. (2017), Matsumoto et al. (2017), Graf et al. (2018)
Astacidins	*Procambarus clarkii*	Broad spectrum antimicrobial		Shi et al. (2014), Roncevic et al. (2020)
Arasin 1 (37 aa)	*Hyas araneus* (spider crab)	Antibacterial		Stensvag et al. (2008)
Bactenicins (5–7 kD)	Bovine neutrophils Sheep and goat leukocytes	Broad spectrum antimicrobial	Protein translation inhibition Binds 70S *T. thermophilus and E. coli*	Gennaro et al. (1989), Shamova et al. (1999), Benincasa et al. (2010), Mardirossian et al. (2014, 2018a), Gagnon et al. (2016), Seefeldt et al. (2016)
BnPRP1 (35 aa–3.8 kD)	*Brassica napus*	Antibacterial (Gram+, Gram−) Broad spectrum antifungal		Cao et al. (2015)
Cg-Prp (37 aa)	*Crassostrea gigas* (oyster)	Antibacterial		Gueguen et al. (2009)
Dolphin Tur1 (32 aa)	*Tursiops truncatus*		Protein translation inhibition Binds ribosome	Mardirossian et al. (2018b)
Drosocins (19 aa)	*Drosophila* Oregon	Antibacterial		Bulet et al. (1993)
Formaecin (16 aa)	*Myrmecia gulosa (red bull ant)*	Antibacterial		Mackintosh et al. (1998)
Metchlnikowin (26 aa)	*Drosophila* Oregon	Antibacterial (Gram+), antifungal		Levashina et al. (1995)
Oncocins (19 aa)	*Oncopeltus fasciatus*	Antibacterial (Gram−)	Protein translation inhibition Binds exit tunnel of 70S *E. coli*	Knappe et al. (2010), Roy et al. (2015), Seefeldt et al. (2015)
P1 to P11 (3–9.5 kD)	*Rapana venosa* hemolymph	Antibacterial (Gram+, Gram−)		Dolashka et al. (2011)
Pr-39	*Sus scrofa*	Multidrug-resistant bacteria		Agerberth et al. (1991), Linde et al. (2001), Gennaro et al. (2002)
Pyrrhocoricins	*Pyrrhocoris apterus*	Antibacterial (Gram−)	Protein translation Inhibition *E. coli*	Cociancich et al. (1994), Kragol et al. (2002), Taniguchi et al. (2016)
AmAMP14	*Antheraea mylita*	Antibacterial, antifungal	Cell membrane damage, cell lysis	Chowdhury et al. (2021)

### Salivary Proteins

Unstructured L-Pro-rich salivary proteins (PRPs) contain up to 40% L-Pro residues and account for ∼70% of total proteins in human saliva (Messana et al., 2015; Lorenzo-Pouso et al., 2018). The acinar cells of parotid and submandibular salivary glands synthesize and secrete acidic (aPRP) and basic (bPRP) proteins (Figure 2). While aPRPs bind calcium and protect the tooth surface, bPRPs bind polyphenols/tannins inducing the astringency sensation that influences diet selection (Canon et al., 2021; Dufourc, 2021). Since tannins induce ER stress and ATF4 expression (Nagesh et al., 2018), and since ATF4 in turn induces the transcription of L-Pro biosynthesis genes (*ALDH18A1* and *PYCR1*) (Han et al., 2013; D’Aniello et al., 2015; Gonen et al., 2019), it is tempting to hypothesize that a neutralizing response axis (ER stress→ATF4→L-Pro biosynthesis→PRP synthesis/secretion) can be induced by tannins in salivary glands.

### Cornified Cell Envelope

Skin is the largest organ of the human body, and it protects internal tissues/organs from water and heat loss, physicochemical insults (e.g., UV light), and microbial attack. Cornifins (or SPRRs) are cross-bridging L-Pro-rich proteins of the cell envelope (Marvin et al., 1992; Steinert et al., 1998a,b), a 5–15 nm thick layer of proteins deposited in epidermis corneocytes (Figure 2). Cornifins are markers of psoriasis syndrome (Luo et al., 2020) and are induced in some tumors (Deng et al., 2020; Sasahira et al., 2021).

### Cell Wall Proteins

The extracellular space in plants and algae contains up to 10% dry weight of hydroxyproline (L-Pro-OH)-rich glycoproteins (HRGPs) such as extensins (Showalter, 1993; Lamport et al., 2011), in which L-Pro-OH constitutes up to 30% of total amino acids (Kieliszewski and Lamport, 1994). Besides being structural pilasters, HRGPs are involved in (i) tissue/organ development (embryo, xylem, pod, root hairs, pollen) (Wu et al., 2001; Velasquez et al., 2011; Ogawa-Ohnishi et al., 2013), (ii) a defense mechanism against environmental stress (heat stress, mechanical wounding and bacterial infection) (Francisco and Tierney, 1990; Zhang et al., 2021b), and (iii) an oxygen barrier in the parenchyma of nitrogen-fixing legume root nodules (nodulins) (Scheres et al., 1990; Sherrier et al., 2005). HRGP synthesis requires free L-Pro, and plants respond to pathogen attack by inducing L-Pro accumulation and HRGP synthesis (Fabro et al., 2004).

## Proline in Energy Provision

**FIGURE 3:**
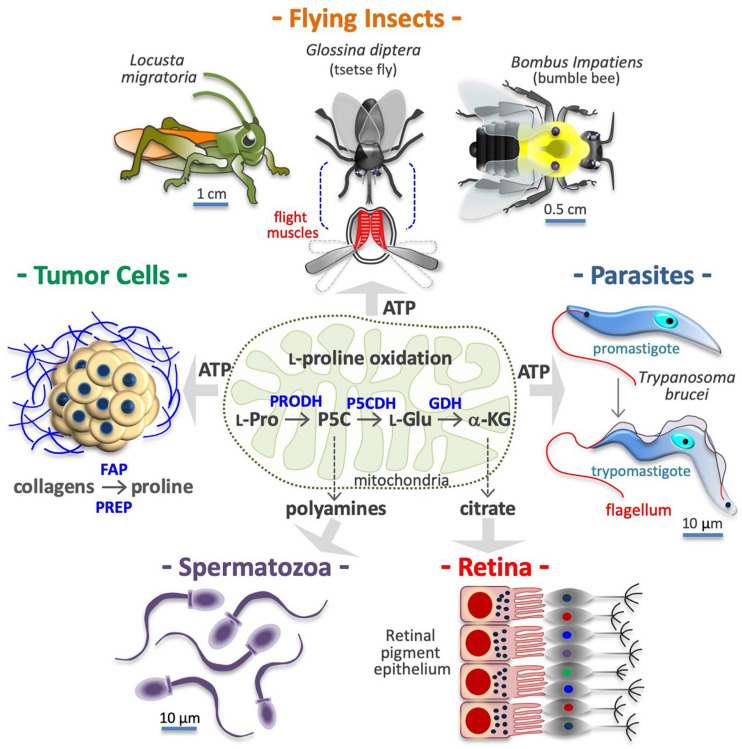
Proline is a source of energy and metabolites. Many cell types obtain ATP/energy from mitochondrial oxidation of proline (*center*), including flight muscle cells of insects **(*top*)**, protozoan parasites **(*middle right*)**, and human cancer cells **(*middle left*)**. Other cells, including motile spermatozoa **(*bottom left*)** use the carbon skeleton of proline to produce polyamines via conversion into pyrroline-5-carboxylic acid (P5C) and ornithine. Lastly, retinal epithelial cells **(*bottom right*)**, produce citrate via conversion of L-proline into L-glutamate (L-Glu) and α-ketoglutarate (α-KG). In the tumor microenvironment, collagens degradation enzymes such as fibroblast activation protein (FAP), and prolyl endopeptidase PREP) release proline-rich peptides and free proline, which after internalization can serves to produce ATP and/or new collagens. Intramitochondrial enzymes involved in L-proline (L-Pro) oxidation, namely proline dehydrogenase (PRODH) and the pyrroline-5-carboxylate dehydrogenase (P5CDH), and glutamate dehydrogenase (GDH), are indicated.

Cells obtain energy/ATP through oxidation of glucose, fatty acids or L-glutamine. However, some cells obtain energy via oxidation of L-Pro in a three-step process (see Figure 3) that converts L-Pro into α-KG, a Krebs cycle intermediate (Tanner et al., 2018). Up to 30 ATP equivalents per L-Pro molecule can sustain the growth of dissimilar cell types, from bacteria to insect muscle cells and human cancer cells (Servet et al., 2012; Nishida et al., 2016). Of note, human genetic defects in L-Pro oxidation are not associated with any developmental deficiency, suggesting that any normal cell type in the human body is strictly reliant on L-Pro energy.

### Cancer Cells

Pancreatic and mammary tumor tissues are full of collagens, providing a large reservoir of free L-Pro (Linder et al., 2001; Barcus et al., 2017). Prolyl-specific peptidases are induced in cancer cells and can release L-Pro-rich peptides and free L-Pro in their microenvironment by degrading ECM collagens (Figure 3; Pure and Blomberg, 2018). For instance, free L-Pro is accumulated in esophageal carcinoma tissue, where it reaches significantly higher levels than in neighboring normal tissues (Sun et al., 2019). Free L-Pro is transported inside cancer cells, where it can be used for anabolic and catabolic purposes. Indeed, PDAC cancer cells (Olivares et al., 2017), colorectal cancer cells (Liu et al., 2012a), and transformed mammary epithelial cells (MCF10A H-Ras^*V*12^) growing as 3D spheroids (Elia et al., 2017) use L-Pro to obtain energy/ATP (Figure 3). L-Pro is also used to produce new collagens (L-Pro recycling), and, eventually, to alter the ECM composition/stiffness (D’Aniello et al., 2020).

### Insect Vectors and Protozoan Parasites

Protozoan parasites adapt their metabolism to the mutable environments encountered throughout their life cycle, including the hemolymph of their insect vectors (Bringaud et al., 2012). *Trypanosoma brucei*, the causative agent of sleeping sickness, is transmitted by tsetse flies (*Glossina diptera*), and both organisms can oxidize L-Pro to accomplish ATP biosynthesis (Figure 3; Michalkova et al., 2014; Mantilla et al., 2017; Smith et al., 2017; Dolezelova et al., 2020; Haindrich et al., 2021; Villafraz et al., 2021). L-Pro sustains *Trypanosoma cruzi* (the causative agent of Chagas disease) cell invasion and intracellular epimastigote-to-trypomastigote transition (Figure 3; Martins et al., 2009; Mantilla et al., 2015; Barison et al., 2017). Parasites also utilize L-Pro for anabolic purposes. For instance, halofuginone, a selective inhibitor of PRS, blocks the synthesis of L-Pro-rich proteins and the proliferation of *Plasmodium falciparum* (the causative agent of malaria) (Hewitt et al., 2017).

### Insect Flight Muscle

Flight is one of the highest ATP/energy-requiring processes in animals, and the muscle cells involved can make use of different energy sources including carbohydrates (e.g., honeybee *Apis mellifera*) and fatty acids (e.g., butterflies) (Bursell, 1975; Candy et al., 1997). Some insects, such as *Locusta migratoria*, *Bombus impatiens* (bumblebee), *Vespula vulgaris* and *Glossina diptera*, oxidize L-Pro to power flight (Figure 3; Teulier et al., 2016). L-Pro supports flight muscle cells of *Aedes aegypti* mosquitoes that feed on blood and can obtain free L-Pro from the hydrolysis of blood proteins and/or from alanine in the fat body (Goldstrohm et al., 2003; Scaraffia and Wells, 2003; Mazzalupo et al., 2016). Indeed, free L-Pro is abundant in the hemolymph of adult female mosquitoes and other insects such as *Diaphorina citri*, the vector of *Candidatus Liberibacter asiaticus* (huanglongbing) (Killiny et al., 2017).

### Polyamine and Citrate Precursors

Some cells use the carbon skeleton of L-Pro to synthesize L-ornithine and L-arginine. For instance, in the gut of neonates, L-glutamate to pyrroline-5-carboxylate conversion is negligible, hence dietary L-Pro is the only source of L-arginine (Tomlinson et al., 2011a,b). In motile human spermatozoa, L-Pro is the precursor of polyamines such as putrescine, spermidine and spermine (Figure 3; Wu et al., 2005, 2008), which are deregulated in hyper-proliferative cancer cells (Bachmann and Geerts, 2018), and thus a potential target for therapeutic anticancer intervention (Murray-Stewart et al., 2016). The three-step L-Pro to α-KG conversion is also activated to generate Krebs-derived metabolic intermediates. For instance, cells of mouse retinal pigment epithelium use L-Pro to synthesize and export citrate, which is consumed by the outer retina (Figure 3; Chao et al., 2017; Yam et al., 2019; Du et al., 2021).

## Proline in Antistress Response

Living cells are subjected to a fluctuating environment involving transient or continuous changes in physicochemical parameters such as temperature, humidity and UV radiation. For instance, humans renal and corneal cells are exposed to discontinuous but substantial variations in osmolality/salinity. To prevent the detrimental effects of such harmful environmental imbalances, cells utilize adaptive mechanisms, including accumulation of highly soluble non-toxic osmolytes and chemical chaperones (protein stabilizers) such as L-Pro. Of course, living cells can tolerate extensive accumulation of L-Pro (up to a 100-fold increase) without suffering of the ionic imbalances induced by accumulation of inorganic osmolytes (e.g., Na^+^, K^+^, Mg^+2^ or Ca^+2^ salts).

### Osmoprotection

**FIGURE 4:**
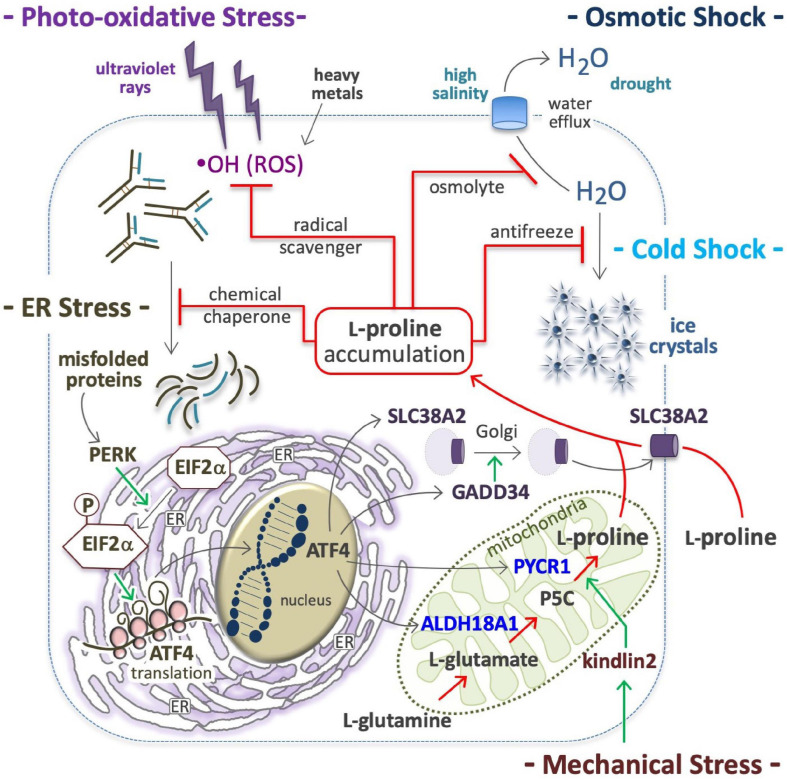
Proline in anti-stress response. Proline accumulation is an evolutionary conserved cell defense mechanism against stressful environments; by quenching hydroxyl radicals (^⋅^OH), protects the cells from ROS oxidations **(*top left*)**; as osmolyte avoids high salinity-mediated cell shrinkage **(*top right*)**, as well as the formation of ice crystal, and thus protects many organisms (yeast, plants, overwinter insect) from cell disruption by freezing **(*middle right*)**. As a chemical chaperone avoids protein denaturation and thus the accumulation of misfolded proteins **(*middle left*)**, which are potent inducers of a molecular response that involves the protein kinase R-like endoplasmic reticulum kinase (PERK), phosphorylation of eukaryotic initiation factor 2 (EIF2), and eventually, the translation of activating transcription factor 4 (ATF4) **(*bottom left*)**; ATF4 in turn, induces the expression of solute carrier family 38 member 2 (SLC38A2), growth arrest and DNA damage-inducible protein (GADD34), aldehyde dehydrogenase 18 family member a1 (ALDH18A1) and pyrroline-5-carboxylate reductase 1 (PYCR1) **(*bottom right*)**. Intracellular proline accumulation through proline uptake and *de novo* proline biosynthesis **(*center*)** can contribute to stress alleviation.

Hypertonic shocks induce water outflow, which reduces the cell volume and lowers macromolecule stability (Burg et al., 2007; Hoffmann et al., 2009; Stadmiller et al., 2017). Cells respond by accumulating L-Pro, which generates an opposite force of water retention (Figure 4). In bacteria, L-Pro accumulation occurs by uptake of extracellular free L-Pro after the induction (up to 700-fold) of a low-affinity L-Pro transporter (Csonka and Hanson, 1991), through degradation of extracellular L-Pro-rich proteins (Zaprasis et al., 2013) and/or *de novo* L-Pro biosynthesis (Patel et al., 2018). The ability to accumulate L-Pro is vital to organisms inhabiting mutable (fresh/brackish water, intertidal) habitats, such as gastropod mollusks (Wiesenthal et al., 2019). Plants respond to drought, salinity and freezing temperatures by accumulating L-Pro (Yoshiba et al., 1997; Szabados and Savoure, 2010; Hnilickova et al., 2021; Papu et al., 2021), and in tomato cells concentrations can reach 60 mM (500-fold higher than normal levels) (Handa et al., 1983). L-Pro accumulation protects human cells from hyperosmotic stress (Thiemicke and Neuert, 2021). Indeed, L-Pro uptake facilitates the recovery a viable cell volume after hypertonic stress (Law, 1991; Bevilacqua et al., 2005; Krokowski et al., 2017), and the PP1 phosphatase subunit protein PPP1R15A/GADD34 promotes *cis*-to-*trans* Golgi trafficking, and the plasma membrane localization of SLC38A2 L-Pro transporter (Figure 4; Krokowski et al., 2017).

### Antifreeze Activity

In yeast, L-Pro accumulation confers ethanol and freezing tolerance (Takagi, 2008). In overwintering insects, L-Pro contributes to water retention and freezing tolerance (Figure 4), and levels increase to ∼80% of the total pool of free amino acids (Kostal et al., 2011, 2016; Rozsypal et al., 2018; Stetina et al., 2018). Of note, hyperprolinemic larvae of the fly *Chymomyza costata* can survive immersion in liquid nitrogen (−196°C) (Kostal et al., 2011). In *Drosophila* larvae, an L-Pro-rich diet increases the whole-body L-Pro concentration (up to 60 mM) and freezing tolerance (Kostal et al., 2012).

### Radical Scavenging

L-Proline protects various human cells such as HEK293, HeLa, HepG2, Jurkat, BJAB, WM35, skin keratinocytes and fibroblasts against ROS-mediated oxidative stress (Figure 4; Wondrak et al., 2005; Krishnan et al., 2008; Natarajan et al., 2012). Of note, the five-membered ring of L-Pro molecule, known as pyrrolidine or tetrahydropyrrole, quenches hydroxyl radicals (^⋅^OH) (Signorelli, 2016). In plants L-Pro accumulates in response to oxidative compounds (Yang et al., 2009; Ben Rejeb et al., 2015), and contributes to protect plants from photo-oxidative stress (i.e., light-dependent generation of ROS) (Liang et al., 2013). Recently, it emerged that salivary L-Pro-rich proteins can neutralize ROS, and specifically hydroxyl radicals (Komatsu et al., 2020).

### Heavy Metal Detoxification

In plants, L-Pro is accumulated after exposure to heavy metals such as cadmium, chromium, and zinc (Sharma et al., 1998; Verbruggen and Hermans, 2008; Hayat et al., 2012; Dubey et al., 2018; Dong et al., 2021; Pejam et al., 2021; Zdunek-Zastocka et al., 2021), and this mitigates the detrimental effects of cadmium in young olive plants (Zouari et al., 2016) and cultured tobacco cells (Islam et al., 2009). Heavy metal toxicity is usually associated with ROS accumulation (Figure 4). Indeed, cadmium induces p53 (Aimola et al., 2012), a transcriptional inducer of PRODH expression (Polyak et al., 1997), which catalyzes L-Pro oxidation in mitochondria, leading to abnormal ROS production and apoptosis (Liu et al., 2006, 2008, 2009; Oscilowska et al., 2021). Thus, a p53→PRODH→ROS→apoptosis axis may be activated as a response to toxic metals such as cadmium.

### ER Stress Relief

At a molecular level, various stressful conditions (e.g., suboptimal temperature, high salinity and oxidative agents) can destabilize the structure and conformation of cellular proteins and other macromolecules. Thus, the accumulation of L-Pro (chemical chaperone) represents a convergent response of cells aimed at inhibiting the formation of unfolded/misfolded protein aggregates. In this context, induction of ATF4 expression (Figure 4), and subsequent enhancement of the transcription of genes involved in L-Pro uptake (*SLC38A2*) and biosynthesis (*ALDH18A2, PYCR1*) can contribute to intracellular L-Pro accumulation (D’Aniello et al., 2015). By stabilizing protein folding and/or promoting protein refolding, L-Pro can avoid and/or relieve ER stress.

## Proline is a Neural Metabotoxin

Schafer et al. (1962) reported a link between hyperprolinemia (HP), characterized by high levels of plasmatic L-Pro, and neuronal dysfunction in human patients. It later emerged that different forms of hereditary human HP (type I or II) are associated with defects in L-Pro oxidation/degradation (Geraghty et al., 1998; Jacquet et al., 2002). Indeed, ectopic expression of PRODH in glioblastoma cells reduces the level of L-Pro (Cappelletti et al., 2018). Free L-Pro can interfere with excitatory presynaptic transmission, and therefore normal neuronal activity in the central nervous system (CNS) (Shafqat et al., 1995; Velaz-Faircloth et al., 1995; Wyse and Netto, 2011). Of note, the psychostimulant methamphetamine induces L-Pro synthesis in human neuroblastoma cells (Jones et al., 2021).

### Schizophrenia Induction and Neurotoxicity

**FIGURE 5:**
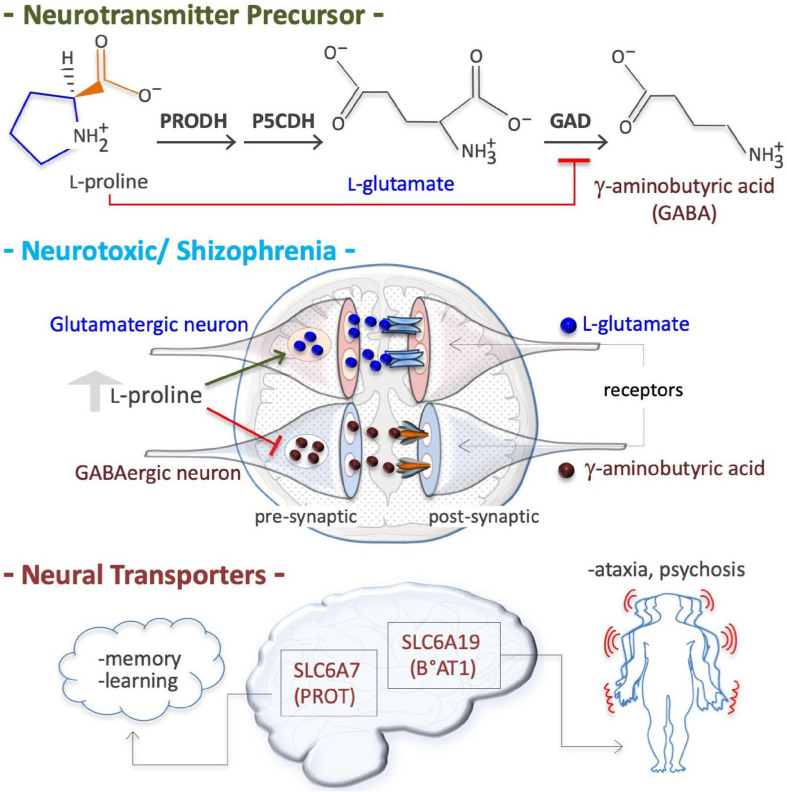
Proline is a neural metabotoxin. Proline is a metabolic precursor of L-glutamate and gamma-aminobutyric acid (GABA), i.e., the two major neurotransmitters in mammalian brain **(*top*)**. At high plasma concentrations (2–3 millimolar instead of 150–200 micromolar), as occurs in patient suffering of hyperprolinemia type II (HPII, Table 3), the neurons can channeled free proline into glutamate biosynthesis, thus increasing free glutamate level. At a high level free proline can inhibit glutamate decarboxylase (GAD) enzyme (GABA biosynthesis) thus reducing GABA level in pre synaptic neurons. Altered levels of both these crucial neurotransmitters, and thus alterations in neurotransmission **(*middle*)**, can explain some of the symptoms of hyperprolinemic patients, including schizophrenia. Defects in neural proline transport, which is mediated by different transporters such as the solute carrier family 6 member 7 (SLC6A7), a high affinity proline transporter, also known as proline transporter 1 (PROT1), and by the solute carrier family 6 member 19 (SLC6A19), also known as system B(0) neutral amino acid transporter 1 (B0AT1), are associated with ataxia and psychosis.

Hyperprolinemia is an etiopathogenetic factor of schizophrenia, a heterogeneous disorder that affects about 21 million people worldwide (Disease et al., 2017). HPI *Drosophila* models (*PRODH* mutants) exhibit a depressed ‘sluggish’ behavior (Hayward et al., 1993), while HPII models (defects in P5C to L-glutamate conversion due to a *P5CDH* mutation) display larval and pupal lethality (He and DiMario, 2011). Conversely, *PRODH*-overexpressing flies exhibit an opposite ‘aggressive’ behavior (Zwarts et al., 2017). HPI mouse models also exhibit sluggish movements (Blake and Russell, 1972; Kanwar et al., 1975) and schizophrenia-related phenotypes (learning, memory and sensorimotor gating) (Gogos et al., 1999; Paterlini et al., 2005). Human patients with genetic defects in *PRODH* (HPI, L-Pro levels up to 10-fold higher than normal) or in *P5CDH* (*ALDH4A1*; HPII, L-Pro levels up to 15-fold higher and P5C excretion) suffer schizoaffective disorders and schizophrenia (Table 2; Liu et al., 2002; Bender et al., 2005; Raux et al., 2007; Clelland et al., 2011; Nagaoka et al., 2020). At high levels, L-Pro can be oxidized/converted into the neurotransmitter L-glutamate, which is associated with schizophrenia (Figure 5). Excess L-glutamate disturbs synaptic transmission and can destroy neurons, a process known as excitotoxicity (Nadler et al., 1988; Cohen and Nadler, 1997). Moreover, acting as a GABA mimetic inhibitor of the GAD enzyme, L-Pro can reduce the synthesis the GABA neurotransmitter, thereby provoking synaptic dysfunction (Figure 5; Crabtree et al., 2016). Of note, L-Pro antagonizes GABA signaling in plants (Haudecoeur et al., 2009).

### Neural Transporters

In neural tissues, two transporters of L-Pro are expressed; solute carrier family 6 member 7 (SLC6A7, PROT), a member of GABA family, and solute carrier family 6 member 19 (SLC6A19, B°AT1) (Figure 5; Roigaard-Petersen and Sheikh, 1984; Malandro and Kilberg, 1996; Thwaites and Anderson, 2007, 2011; Verrey et al., 2009). Genetic and/or pharmacological inhibition of SLC6A7 reduces locomotor activity and improves mouse learning and memory (Zipp et al., 2014; Schulz et al., 2018). SLC6A7 is induced in fibroblasts of patients suffering of Friedreich’s ataxia, characterized by a lack of control in muscle activity/movements (Napierala et al., 2017). Mutations of *SLC6A19* are associated with Hartnup disease, a complex syndrome involving cerebellar ataxia and psychosis (Seow et al., 2004). *SLC6A20* (IMINO) is expressed in human neurons and regulates L-Pro and glycine homeostasis (Bae et al., 2021).

### Neural Bioactive Peptides

Collagen-derived peptides such as Pro-Pro-OH induce the expression of crucial neural growth factors in the hippocampus of mice, increasing both dopamine concentration in the prefrontal cortex and proliferation of neural progenitor cells, and, eventually, reducing depression-like behavior (Mizushige et al., 2019; Nogimura et al., 2020). L-Pro-containing peptides (Gly-Pro-Glu and cyclo-Gly-Pro) inhibit inflammation and induce vascular remodeling, thereby protecting brain tissues from ischemic injury (Guan and Gluckman, 2009). Moreover, a phosphine analog of Pro-Gly-Pro tripeptide displays neuroprotective properties (Alexey et al., 2021).

### Leukodystrophy/Cerebral Hypomyelination

Genetic defects in *PYCR2*, a *PYCR1* paralog, are associated with leukodystrophy-hypomyelinating 10 (HLD10; Table 2), a syndrome characterized by microcephaly and psychomotor disability (Nakayama et al., 2015; Zaki et al., 2016). PYCR2-deficient fibroblasts derived from HLD10 patients are highly susceptible to oxidative stress-induced apoptosis, and this may contribute to this complex phenotype (Reversade et al., 2009; Nakayama et al., 2015).

## Proline Modulates Signaling Pathways

The availability of some amino acids influences the activity of cell signaling pathways. For instance, the level of L-glutamine, L-leucine, and L-arginine impacts the mechanistic target of rapamycin (mTOR) pathway (Curi et al., 2007; Xie and Klionsky, 2007; Ryter et al., 2013; Bar-Peled and Sabatini, 2014; Lahiri et al., 2019). L-tyrosine and L-phenylalanine modulate the G protein-coupled receptor 142 (GPR142)-mediated pathway (Lin et al., 2016). It emerged that mESCs, isolated from mouse blastocysts, suffer from a finely regulated partial shortage of L-Pro, and that an increase in free L-Pro availability modulates the activity of the amino acid stress response (AAR), fibroblast growth factor/extracellular signal-related kinase (FGF/ERK), TGFβ, wingless and int-1 (WNT), and redox signaling pathways. As expected, specific signaling modulators such as halofuginone (AAR inducer), SB431542 (TGFβ inhibitor), CHIR99021 (WNT agonist) and PD0325901 (MEK/ERK inhibitor) fully counteract L-Pro supplementation effects (Comes et al., 2013; D’Aniello et al., 2015). Moreover, L-Pro impacts mTOR pathway in porcine trophectoderm cells (Liu et al., 2021).

### Amino Acid Starvation Response

**FIGURE 6:**
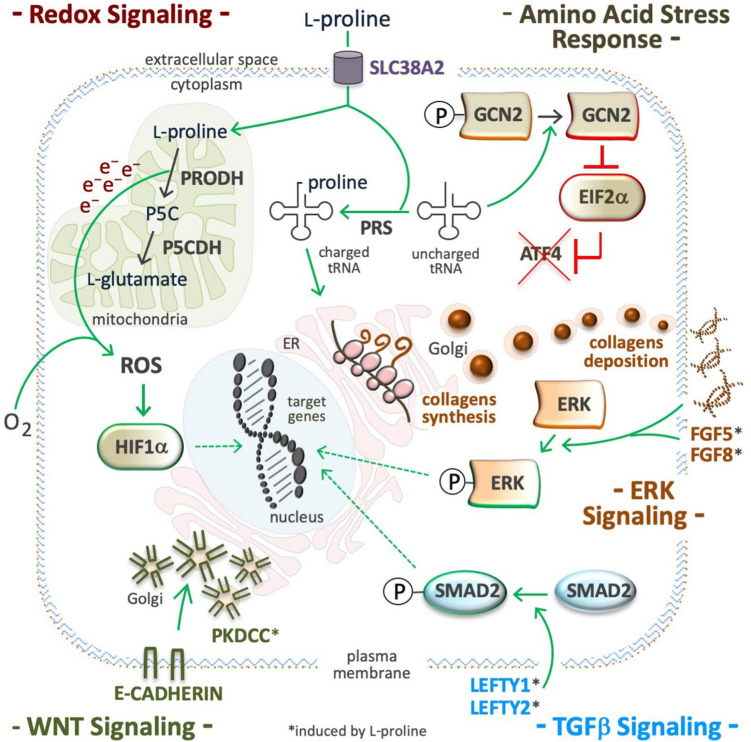
Proline modulates signaling pathways. Supplemental proline, after being transported into the cell cytoplasm can be used and/or for mitochondrial oxidation (catabolism) and/or for the loading of uncharged tRNA (anabolism). ROS are byproducts of proline oxidation **(*top left*)** and their accumulation can stabilize the hypoxia inducible factors (HIF), and modulate redox signaling **(*middle left*)**. Proline-tRNA loading induces dephosphorylation (inactivation) of the general control non-repressible 2 (GCN2) kinase, also known as eukaryotic translation initiation factor 2 alpha kinase 4 (EIF2AK4). The inactive form of GCN2 is unable to phosphorylate eukaryotic translation initiation Factor 2A (EIF2A), and to promote translation of activating transcription factor 4 (ATF4), so inactivating the amino acid stress response (AAR). Proline-tRNA loading also improves collagens expression, which are modulators of the integrin linked kinase/extracellular signal-regulated Kinase (ILK/ERK) super-pathway **(*middle right*)**. Proline abundance induces left-right determination factors (LEFTY1, LEFTY2), the phosphorylation of mothers against decapentaplegic homolog 2 (SMAD2), and thus, the activation of the transforming growth factor beta signaling pathway **(*bottom right*)**. Proline abundance also induces the expression of the protein kinase domain containing, cytoplasmic (PKDCC), and the delocalization the E-cadherin from plasma membrane to *trans* Golgi **(*bottom left*)**.

In cultured ESCs, exogenously available L-Pro, at a physiological concentration range (50–250 μM), disables the AAR pathway by improving L-Pro-tRNA loading, inactivating (dephosphorylation) eukaryotic translation initiation factor alpha (EIF2α), and eventually, preventing translation of ATF4 mRNA (Figure 6; D’Aniello et al., 2015). In the absence of ATF4, the genes involved in L-Pro biosynthesis (*ALDH18A1* and *PYCR1*), and L-Pro uptake (*SLC38A2* and *GADD34*) are silenced (Gaccioli et al., 2006; D’Aniello et al., 2015). L-Pro-ATF4 interplay also impacts cardiac fibroblast metabolism (Qin et al., 2017). Human kidney and breast cancer cells suffer from a similar intrinsic and partial shortage of L-Pro (Loayza-Puch et al., 2016; Sahu et al., 2016).

### Extracellular Signal-Regulated Kinase

In stem and cancer cells, a high L-Pro regimen induces phosphorylation of ERK1 and enhances the transcription of ERK-related genes (Figure 6; Liu et al., 2006; D’Aniello et al., 2016). Supplemental L-Pro induces the expression of growth factors (FGF5, FGF8, and FGF13) and the synthesis of collagen, and this can contribute to the induction of the ILK/ERK super-pathway, as revealed by transcriptome analysis (Comes et al., 2013; D’Aniello et al., 2016, 2019b). Indeed, collagen mimics consisting of repeated units (5 or 10) of the Pro-Pro-Gly tripeptide activate phosphoinositide 3-kinase (PI3K)-dependent p38 mitogen-activated protein kinase (MAPK) phosphorylation (Weinberger et al., 2005).

### Transforming Growth Factor

In ESCs, supplemental L-Pro induces expression of left-right determination factors (LEFTY1 and LEFTY2) and phosphorylation (activation) of small mother against decapentaplegic (SMAD2), which are extracellular inhibitors and intracellular effector of TGFβ-signaling, respectively (Figure 6; D’Aniello et al., 2015, 2016). In VSMCs of injured arteries (Majesky et al., 1991; Ensenat et al., 2001), and in meniscal fibrochondrocytes (Pangborn and Athanasiou, 2005), supplemental TGFβ induces L-Pro uptake and collagen deposition. A L-Pro→TGFβ→L-Pro regulatory loop should allow the induction of collagen synthesis only when free L-Pro is sufficient to warrant timely tRNA loading, thus avoiding ribosome stalling (ER stress).

### Wingless and Int-1

Pluripotent stem cells tend to proliferate as tightly packed cell aggregates, a trend that is inverted by a high L-Pro regimen (Comes et al., 2013). This phenotypic effect of L-Pro is fully counteracted by CHIR99021, a WNT signaling agonist. Moreover, L-Pro abundance delocalizes E-cadherin from the plasma membrane, where it is involved in cell-cell adherent junctions, to the Golgi. This subcellular redistribution of E-cadherin relies on the protein kinase domain containing, cytoplasmic (PKDCC), also known as vertebrate lonesome kinase (VLK) (Figure 6; Comes et al., 2013). L-Pro supplementation induces the expression of insulin-related genes such as *IGF2*, *IGFR1*, *IGFBP3*, *IRS1* and *IRS2* (D’Aniello et al., 2016), which are modulators of glycogen synthase kinase 3 (GSK3) activity (Desbois-Mouthon et al., 2001), and enhanced translation of collagen XVIII, which contains a frizzled-like domain (Heljasvaara et al., 2017), and can contribute to WNT modulation.

### Autophagy

In mouse ESCs, L-Pro supplementation enhances L-Pro-tRNA loading and inhibits autophagy. Accordingly, halofuginone inhibits L-Pro-tRNA loading and activates autophagy (D’Aniello et al., 2015). In human and murine ECSLC, knockdown of Tap73 tumor protein reduces L-Pro biosynthesis and induces autophagy (Sharif et al., 2019). Protracted exposure to free L-Pro induces stem cell motility, invasiveness, and macro-autophagy (D’Aniello et al., 2015). In cancer cells overexpressing PRODH and exposed to a high exogenous L-Pro regimen, autophagy is induced (Liu et al., 2012b).

### Reactive Oxygen Species and Hypoxia-Inducible Factors

Electrons released during mitochondrial L-Pro oxidation reduce flavin adenine dinucleotide (FAD) to generate FADH2 and/or O_2_ during the production of ROS (Figure 6; Donald et al., 2001). In *Arabidopsis thaliana*, PRODH-mediated production of sub-lethal levels of ROS induces disease resistance (Cecchini et al., 2011), and in *Caenorhabditis elegans* this prolongs the nematodes life span (Zarse et al., 2012). In *C. elegans*, defects in L-Pro catabolism results in premature reproductive senescence and male infertility (Yen and Curran, 2021). In cancer cells, the L-Pro->PRODH->ROS axis can activate either pro-tumorigenic (cell survival) or anti-tumorigenic (cell death) signaling (Moloney and Cotter, 2018; Oscilowska et al., 2021). In rats’ blood cells, hyperprolinemia increases oxidative damage of proteins, lipids and DNA (Ferreira et al., 2014). The effect of L-Pro on intracellular redox balance can be amplified by an NADPH-consuming futile cycle of L-Pro/P5C inter-conversion (Phang, 2019). Besides ROS, oxidative deamination of L-Pro generates α-KG, an essential substrate for hydroxylating dioxygenase enzymes, including PHD1-3 enzymes that catalyze the post-translational hydroxylation of specific proline residues of hypoxia-inducible factors (HIFs) resulting in destabilization of the protein. Indeed, the induction of PRODH activity in cancer cells destabilizes HIF1α and down-regulates the transcription of HIF1α target genes (Liu et al., 2009).

## Proline is an Epigenetic Modifier

**FIGURE 7:**
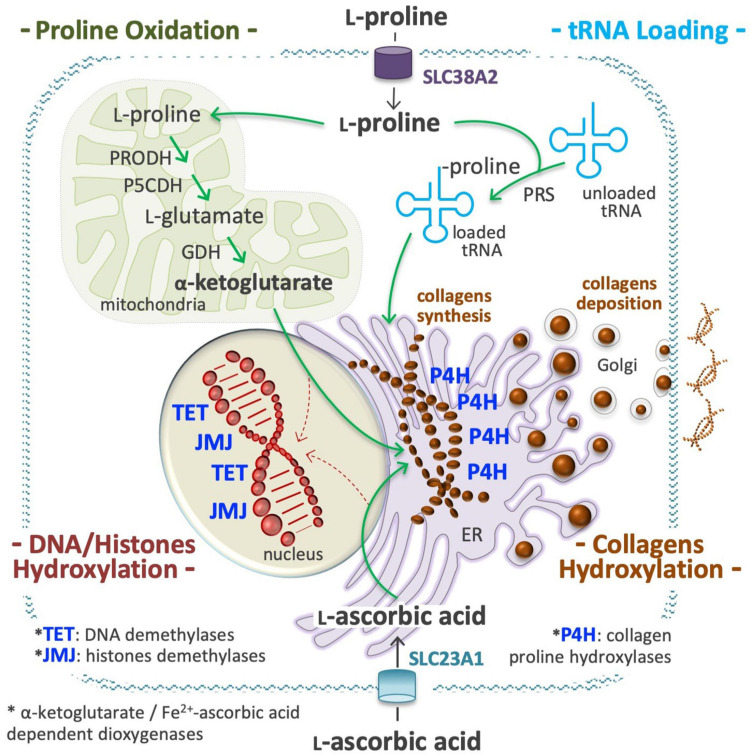
Proline is an epigenetic modifier. At a high proline regimen, extracellular proline is channeled into the cell cytoplasm through a transport system, as the solute carrier family 38 member 2 (SLC38A2), also known as system N amino acid transporter 2 (SNAT2), and used to charge tRNA molecules **(*top right*)**, in a reaction catalyzed by the prolyl-tRNA synthetase (PRS). A high level of charged Proline-tRNA is an essential requisite for collagens synthesis **(*middle*)**. A high fraction of L-Pro residues of the nascent molecules of collagens are hydroxylated by prolyl 4-hydroxylases (P4H 1, 2, 3) dioxygenases enzymes, a process that consume huge amounts of ascorbic acid (vitamin C, VitC) and α-ketoglutarate (α-KG) **(*middle right*)**. VitC is transported by members of the solute carrier family 23 (SLC23A1, 2; ***bottom***), whereas α-KG is produced inside mitochondria using proline and/or glutamate as precursors **(*top left*)**. A sudden and sizeable increment of P4H activity in the endoplasmic reticulum (ER) can reduce the availability of VitC and α-KG for the activity of nuclear dioxygenases involved in DNA methylcytosine hydroxylation/demethylation (ten-eleven translocation, TET 1, 2, 3) and in histones lysine hydroxylation/demethylation (jumonji, JMJ) **(*bottom left*)**. This compartmentalized metabolic perturbation, by increasing the DNA and histones methylation levels, can modify the epigenetic landscape of the cells.

Several metabolites may influence, directly or indirectly, the activity of chromatin-modifying enzymes, and thus the epigenetic landscape of the cells (Reid et al., 2017; D’Aniello et al., 2019b; Surguchov et al., 2021). L-Pro is not a substrate, product, cofactor, or allosteric regulator of any epigenetic enzyme, but in ESCs its availability influences the activity of ten-eleven translocation (TET; DNA) and Jumonji (JMJ, histone) demethylase enzymes, which are strictly dependent on the availability of O_2_, α-KG, and ascorbic acid (vitamin C, VitC) to be active (Figure 7; Comes et al., 2013; D’Aniello et al., 2016, 2019b).

### DNA Methylation

L-Proline supplementation increases DNA 5-methylcytosine (5mC) and reduces 5-hydroxy-methylcytosine (5hmC) levels, inducing ∼1 × 10^3^ DMRs distributed throughout all chromosomes of ESCs, with ∼50% of DMRs located in gene promoter regions (mostly H) and ∼20% in gene enhancers (D’Aniello et al., 2016). Importantly, ∼95% of genome sites hypermethylated after L-Pro supplementation are hypomethylated following VitC (50–150 μM) supplementation, indicating that L-Pro and VitC induce opposite epigenetic alterations in the same DNA regions. VitC is needed for the activity of TET demethylases (Blaschke et al., 2013), and ∼90% of genomic regions hypermethylated in by a high L-Pro regimen are hypermethylated also in cells lacking TET-mediated DNA demethylase activity (Lu et al., 2014; D’Aniello et al., 2019a).

### Histone Methylation

L-Proline supplementation also triggers a genome-wide reprogramming of H3K9 methylation status, altering more than 1.6 × 10^4^ genome sites located mainly in non-coding intergenic regions (Comes et al., 2013). Demethylation is catalyzed by members of the JMJ dioxygenase enzyme family, and upon silencing of Jmjd1a (H3K9 demethylase), ESCs adopt a molecular (upregulation of *Fgf5* and *Brachyury* genes) and phenotypic (irregular flat-shaped colonies, sensitivity to trypsin digestion) state of pluripotency, similar to that induced by a high L-Pro regimen (Loh et al., 2007). Differences in the expression level and/or in the kinetic parameters (substrate affinity) of different JMJs can explain how L-Pro abundance alters the methylation level of some specific lysine residues (K9, K36) of histone H3.

### Metabolic Imbalance

It recently emerged that a sudden and substantial increase in L-Pro stimulates collagen synthesis in the ER of ESCs (D’Aniello et al., 2019a), and that a significant fraction of L-Pro residues of nascent collagens are hydroxylated by prolyl 4-hydroxylase (P4H) dioxygenases, in particular by P4HA1 and P4HA2 enzymes, with depletion of α-KG and VitC. Under such conditions, nuclear dioxygenases such as TETs and JMJs lose activity, and consequently, DNA and histone methylation levels increase (Figure 7). Genetic and pharmacological evidence supports the idea that an abrupt induction of collagen synthesis leads to a similar metabolic imbalance and epigenome alterations also in cancer cells (D’Aniello et al., 2019a).

## Proline Induces Proliferation of Stem and Tumor Cells

Pluripotent stem cells shape the ICM in blastocysts of mammals and the apical meristems of plant organs (shoots and roots), and can self-renew and undergo differentiation into various somatic lineages. Cancer cells often display a stem cell-like growth behavior. Of note, L-Pro is a growth limiting metabolite (intrinsic starvation) for embryonic stem cells (D’Aniello et al., 2015), and for many different human cancer cells (D’Aniello et al., 2020). Similarly, L-Pro metabolism also influences the proliferation of meristematic and plant tumor cells (Trovato et al., 2001; Biancucci et al., 2015).

### Stem Cells

**FIGURE 8:**
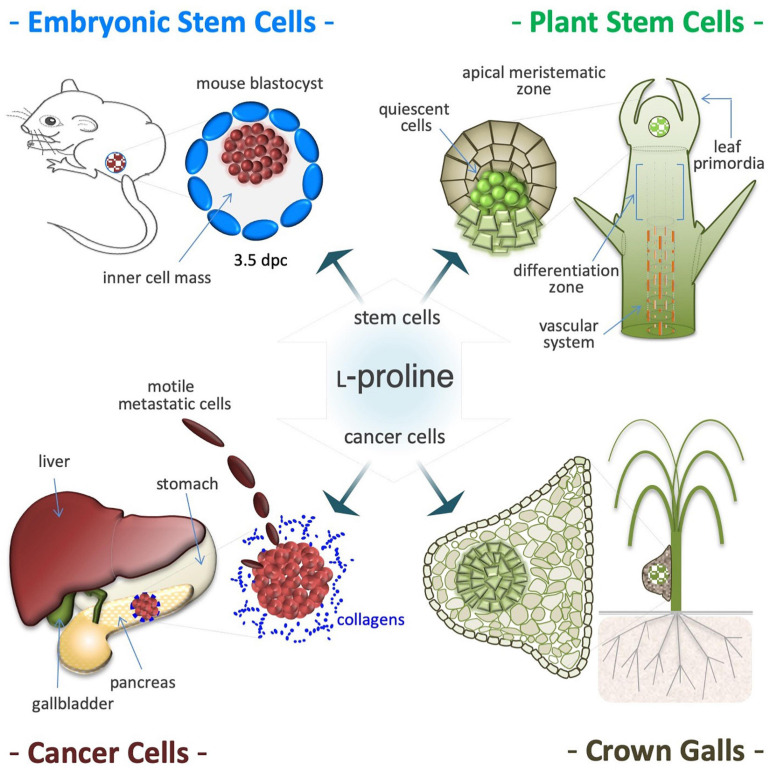
Proline induces proliferation of stem and cancer cells. Proline availability controls proliferation of pluripotent embryonic stem cells (ESCs), isolated from the inner cell mass of 3.5 days post coitum (dpc) mouse blastocysts **(*top left*)**. Proline induces proliferation also of plant stem cells that shaped the shoot and root apical meristems and are responsible of post-embryonic organogenesis **(*top right*)**. Proline improves proliferation and invasiveness of cancer cells primarily by increasing protein synthesis **(*bottom left*)**. Deregulation of proline metabolism is involved in development and growth of plant tumors, such as the neoplastic hairy roots and are tumor-like structures known as crown galls. Usually, these tumors are formed in the roots or in the lower stem region **(*bottom right*)**, and display a single and/or several centers of deregulated cell proliferation (hyperplasia), surrounded by hypertrophic tissues **(*bottom right*)**.

Supplemental L-Pro (50–250 μM) improves proliferation of ESCs (Figure 8; Washington et al., 2010; Casalino et al., 2011), development of pre-implantation embryos (Morris et al., 2020) and fetus survival (Liu et al., 2019). L-Pro is internalized into stem cell cytoplasm through the SLC38A2 (SNAT2) transporter (Tan et al., 2011), and halofuginone (prolyl-tRNA synthetase inhibitor) fully counteracts L-Pro induction of cell proliferation (D’Aniello et al., 2015). Moreover, halofuginone and L-Pro modify the ESC transcriptome in opposite directions (D’Aniello et al., 2015), showing that mouse ESCs are partially starved of L-Pro, even after incubation in complete rich medium. Of note, during *in vitro* fertilization of mouse oocytes, L-Pro supplementation improves stem cells (ICM) proliferation and embryo development (Treleaven et al., 2021).

### Cancer Cells

L-Proline shortage is a major cause of partial ribosome stalling (diricore analysis) suffered by kidney and breast cancer cells (Loayza-Puch et al., 2016). Likewise, up-regulation of L-Pro biosynthesis genes (*ALDH18A1* and *PYCR1*) also reveals L-Pro starvation in tumor cells (D’Aniello et al., 2020). Moreover, *ALDH18A1* knock-down activates AAR stress signaling, and reduces melanoma tumor growth both *in vitro* and *in vivo* (Kardos et al., 2015), whereas *PYCR1* induction improves proliferation and invasiveness of breast, esophagus, lung, melanoma, pancreas, and prostate cancer cells (Nilsson et al., 2014; Ding et al., 2017; Zeng et al., 2017; Cai et al., 2018; Ye et al., 2018; Kardos et al., 2020; Forlani et al., 2021). Of note, kindlerin 2 (KINDLING-2) protein stabilizes the mitochondrial PYCR1 enzyme, increasing L-Pro synthesis and lung adenocarcinoma cell proliferation (Guo et al., 2019). Importantly, translocation of KINDLING-2 into mitochondria is regulated by ECM stiffness (Guo et al., 2019) and PINCH-1 (particularly interesting new Cys-His protein 1) protein (Guo et al., 2020; Ding et al., 2021), and PYCR1 activity is modulated by the mitochondrial deacetylase sirtuin (SIRT3) (Chen et al., 2019). PYCR1 stabilization by KINDLING-2 induces L-Pro synthesis in human lung fibroblasts and contributes to pulmonary fibrosis progression (Zhang et al., 2021a).

### Meristematic Cells

Post-embryonic organogenesis in adult plants relies on apical meristems, and a fine-tuned balance between self-renewal and differentiation fates adapts organ morphogenesis to a fluctuating environment (Figure 8; Mattioli et al., 2009; Lehmann et al., 2010; Szabados and Savoure, 2010). In *Arabidopsis*, L-Pro availability controls root meristem activity (Biancucci et al., 2015) by modifying the expression of L-Pro-rich proteins, and regulating a compartmentalized (mitochondria/cytoplasm) cycle of L-Pro synthesis and degradation that modifies the NADP^+^/NADPH ratio (Verslues and Sharma, 2010). Therefore, it is tempting to hypothesize that the induction of L-Pro accumulation during osmotic shock (see Figure 4), by altering the behavior/fate of stem cells, can contribute to couple a harmful environment (soil wetness) with the induction of organogenesis (root elongation).

### Neoplastic Hairy Roots

L-Proline metabolism and plant tumor development are linked by the *rolD* gene of *Agrobacterium rhizogenes*, which encodes OCD that catalyzes L-Orn to L-Pro conversion, and is essential for the induction of neoplastic hairy roots (Figure 8; White et al., 1985; Costantino et al., 1994; Trovato et al., 2001). L-Pro accumulates in root tumor-like galls induced by the nematode *Meloidogyne javanica* or by *Agrobacterium tumefaciens* (Wachter et al., 2003; Trovato et al., 2018). Importantly, bacteria-induced tumorigenesis is attenuated in transgenic plants with low L-Pro levels (Haudecoeur et al., 2009).

## Proline Controls Cell Plasticity

Some metabolites modulate relevant phenotypic transformations such as stem cell differentiation, somatic cell reprogramming, and EMT. For instance, butyric acid drives the differentiation of MSCs into adipocytes (Tugnoli et al., 2019), and, conversely, enhances the reprogramming efficiency of fetal fibroblasts into pluripotent cells (Liang et al., 2010; Mali et al., 2010). Likewise, VitC improves cell differentiation (Cao et al., 2012) and reprogramming (Esteban et al., 2010). Similarly, L-Pro governs the morphology, migratory behavior and pluripotency state of stem cells (Washington et al., 2010; Casalino et al., 2011).

### Cytoskeletal Rearrangements

**FIGURE 9:**
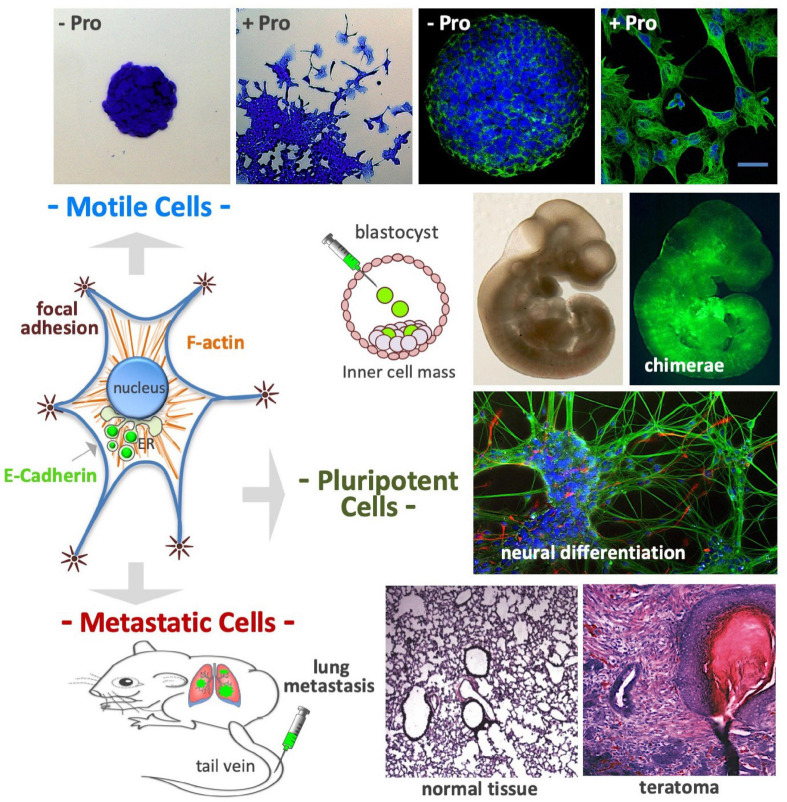
Proline controls cell plasticity. After proline supplementation (50–150 μM) cultured embryonic stem cells undergo a fully reversible phenotypic transition named embryonic stem-to-mesenchymal transition (esMT); after 3–4 days of incubation the cells become motile, spread out from the cell colony core **(*top left*)**, and display complex cytoskeleton rearrangements, with prominent actin stress fibers and focal adhesions **(*top right*)**. The E-cadherin protein, which is an essential component of cell-cell adherens junctions, is delocalized from the plasma membrane to the Golgi complex **(*middle left*)**. Even after acquiring mesenchymal features, the stem cells retain a high degree of pluripotency: (i) colonize mouse embryos after being injected in the inner cell mass of developing mouse blastocysts **(*middle*)**; (ii) undergo *in vitro* neuronal differentiation **(*middle*)** and; (iii) after being injected in the tail vein of mouse **(*bottom left*)**, the cells reach the lungs, and after extravasation, generate metastatic teratomas, which display histologically noticeable differences from normal lung tissue **(*bottom right*)**.

Embryonic stem cells seeded at a low density (50–250 cells/cm^2^) in a high L-Pro regimen develop flat-shaped cell colonies formed by a core of adherent cells surrounded by a crown of detached cells showing mesenchymal features such as long actin stress fibers and mature focal adhesion complexes (Figure 9; Casalino et al., 2011; Comes et al., 2013). These L-Pro-induced cells are in a ‘metastable’ equilibrium, spread out from the colony core and rapidly moving back to re-establish adherent cell-cell contacts, a fully reversible phenotypic transition known as embryonic stem cell-to-mesenchymal transition (esMT) (Comes et al., 2013). Of note, in detached cells, E-cadherin is delocalized from the plasma membrane to the Golgi (see Figure 6) and unlike canonical EMT, during esMT the *CDH1* gene is not down-regulated (Comes et al., 2013).

### Invasion/Metastasis

After exposure to a high L-Pro regimen, ESCs acquire the ability to migrate through matrigel-coated porous membranes in response to serum gradients, or toward chemo-attractants such as EGF and stromal cell-derived factor 1 (Comes et al., 2013). These cells are able to reach the lung tissues after intravenous injection, and to generate tumors with a histological complexity of teratomas (Comes et al., 2013). Thus, a high L-Pro regimen converts adherent stem cells into spindle-shaped, motile and metastatic stem cells (Figure 9).

### Metabolic Reprogramming

The morphological changes induced by L-Pro supplementation are associated with a metabolic switch from a bivalent to a more glycolytic metabolism. Indeed, metabolome profile analysis revealed higher lactate levels and increased susceptibility to 2-DG, a specific inhibitor of the glycolytic pathway (D’Aniello et al., 2016). Moreover, a high L-Pro regimen reduces the mitochondrial membrane potential, which relies on oxidative phosphorylation rates (D’Aniello et al., 2017), thus supporting glycolytic energy metabolism.

### Pluripotency

L-Proline supplementation remodels the transcriptome of naïve ESCs by altering the expression of ∼1.5 × 10^3^ protein-coding genes mainly related to cell adhesion, cell junction, and cell motility functions (Comes et al., 2013; D’Aniello et al., 2017). Cells treated with L-Pro are leukemia inhibitory factor (LIF)-dependent, express pluripotency markers as Nanog homeobox, can differentiate into cardiomyocytes and neurons *in vitro*, and are able to colonize mouse blastocysts (chimeric embryos; Figure 9; Casalino et al., 2011). Recently, Cermola et al. (2021) reported that L-Pro-treated ESCs can differentiate into primordial germ cell like cells (PGCLCs), and are competent to develop elongated gastruloids, suggesting that L-Pro abundance drives ESCs into an early primed state of pluripotency.

### Proline Antagonists

L-Proline-induced esMT is inhibited by well-known chemical modulators of key signaling pathways such as CHIR99021 (WNT agonist) and PD0325901 (TGFβ antagonist) (D’Aniello et al., 2016). Moreover, D’Aniello et al. (2019a) made use of the cell colony morphology to develop a high-throughput screening method, and identified 14 FDA-approved drugs (from 1200 assayed) able to inhibit esMT without preventing L-Pro-induced cell proliferation. Spiramycin (macrolide), Propafenone (flavonoid) and Budesonide (steroid) inhibit esMT and have very different chemical structures, implying molecular complexity in L-Pro-mediated control of stem cell plasticity. Importantly, VitC, but not other antioxidants such as NAC, is a full inhibitor of esMT (D’Aniello et al., 2016).

## Conclusion and Perspectives

**FIGURE 10:**
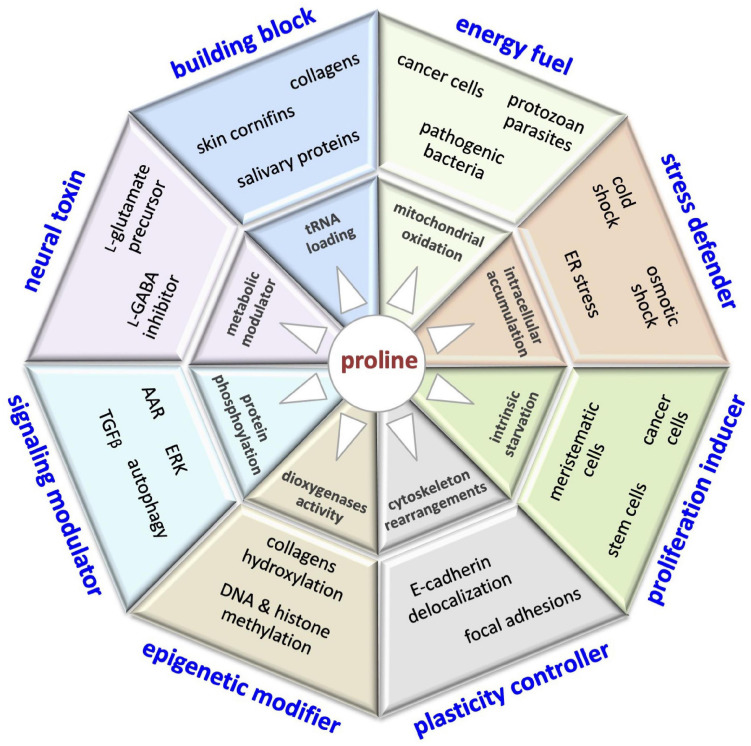
This illustration encompasses the multifaceted roles of proline in cell biology, including: building block of proteins (collagens, cornifins, salivary proteins); energy fuel (bacteria, parasites, cancer cells); stress defender (cold shock, drought/salinity, ER stress); proliferation inducer (stem, meristematic, cancer cells); plasticity controller (cell shape, motility); epigenetic modifier (DNA/histone methylation); modulator of cell signaling pathways (AAR, autophagy, ERK, TGFβ) and neural toxin associated (schizophrenia).

The control of L-Pro metabolism in human cells is relatively poorly understood, even though it might have a great impact on human health (Figure 10). For instance, PrAMPs displaying potent antimicrobial activity and low toxicity for human cells could be efficient tools to fight multidrug-resistant pathogens, a serious public health concern (Charon et al., 2019). Salivary proline-rich peptides able to neutralize microbe attacks could contribute to avoiding the development of dental caries, an infectious disease that affects billions of people (Werneck et al., 2010; Stromberg et al., 2017). Moreover, salivary proteins could contribute to food choices, and so to nutrition status and health (Melis et al., 2021). Translational suppression of proline-rich proteins by pharmacological targeting of the PRS is emerging as an attractive therapeutic approach for the treatment of different diseases. Of note, halofuginone, a specific inhibitor of the PRS, is already in clinical trials for the treatment of fibrotic diseases (Pines and Spector, 2015), and has been recently shown to inhibit SARS-CoV-2 infection, suppressing the translation of proline-rich host attachment factors (Sandoval et al., 2021).

Exploitation of L-Pro as a source of carbon and/or energy appears to be an adaptive response of cells to high-L-Pro microenvironments, which can be generated by pathological tissue damage (bacterial invasion, cancer progression, trauma). Although never measured, it is possible to speculate that in an extremely confined extracellular space, free L-Pro can reach exceptionally high concentrations. L-Pro supports invasiveness of bacteria, parasites and cancer cells, all processes that engage tissue degradation/remodeling (Christgen and Becker, 2019; D’Aniello et al., 2020), and D-Pro-derived peptidomimetic inhibitors of human gelatinases/metalloproteinases involved in tissue remodeling are potential anti-metastatic agents (Lenci et al., 2021). Moreover, enzymes involved in L-Pro metabolism are potential targets of antiparasitic drugs (Saye et al., 2017; Ugwu et al., 2018).

Various stressful conditions, including suboptimal temperature, high salinity and oxidative agents, can alter the conformations of proteins and other macromolecules. Since L-Pro is a potent and non-toxic chemical chaperone, its intracellular accumulation could be an evolutionarily conserved response aimed at inhibiting the formation of unfolded/misfolded protein aggregates. Indeed, hemocompatible gold nanoparticles coated with L-Pro inhibit both collagen fibril formation (Anand et al., 2017) and insulin aggregation (Prajapati et al., 2021), and could provide a basis for creating antifibrotic and antiamyloid formulations.

Numerous studies conclude that at high levels, free L-Pro is a neurotoxin. Lactic acid inhibits PRODH activity, and lactic acidosis syndrome (blood lactic acid >5 mM) is frequently associated with hyperprolinemia, supporting the idea that in adult humans L-Pro homeostasis is strictly dependent on L-Pro oxidation. Of note, L-Pro at high levels is harmful for brain/neural activity, but acting as a chemical chaperone it can prevent protein unfolding/misfolding (Liang et al., 2014). Thus, regulation of L-Pro metabolism is studied in the context of neurodegenerative diseases associated with the formation of protein aggregates, as exemplified by Huntington’s, Parkinson’s, and Alzheimer’s (Powers et al., 2009; Khan et al., 2010).

Beyond some cancer cells, whether and which normal human cells oxidize L-Pro, and whether this contributes to maintain prolinemia, remains unknown. The concomitant activation of L-Pro oxidation (for ATP production in mitochondria) and tRNA loading (for collagen synthesis in the ER) remains uncharacterized at the single-cell level. By generating sublethal amounts of ROS, L-Pro oxidation can induce redox signaling, and eventually a compensatory stress response, through the induction of ROS consuming/neutralizing enzymes. Importantly, in bacteria (Zhang et al., 2015), fungi (Chen and Dickman, 2005) and nematodes (Zarse et al., 2012), L-Pro oxidation increases cell resilience to stressful conditions. However, the induction of stress tolerance by L-Pro oxidation in human cells remains an open question.

Aging is usually associated with a significant reduction (quantitative and qualitative) in CTs (tendon, bone, cartilage), for which L-Pro is essential. Of note, older people and patients suffering hereditary defects L-Pro biosynthesis share a similar aged appearance (e.g., osteopenia, cataracts, wrinkled skin, *cutis laxa*). Furthermore, sedentary life-induced sarcopenia is associated with hyperprolinemia, but its impact on neural disorders suffered by the elderly is unknown.

How L-Pro availability modulates stem and cancer cell proliferation is an interesting question that is getting increasingly attention. Free L-Pro can improve the translation of L-Pro-rich proteins (Sabi and Tuller, 2015; Chyzynska et al., 2021) or simple protein stretches, as demonstrated for HOXB4 involved in leukemia (Cusan et al., 2017). Recently, cell-based drug screening identified 137 drugs (out of 1200 assayed) able to inhibit stem cell proliferation, of which 80% also inhibited cancer cells (D’Aniello et al., 2019a), suggesting a similar chemosensitivity spectrum. Thus, the development of therapeutic strategies to target L-Pro metabolism may provide new options to eradicate cancer cells. Importantly, L-Pro abundance induces invasiveness in stem cells, a peculiar trait of migrating cancer cells. Certainly, the ability of L-Pro to control morphogenesis is not limited to stem cells. For instance, L-Pro availability influences plant shoot and root development (see Biancucci et al., 2015, for a review), hyphal morphology in the pathogenic fungus *Colletotrichum trifolii* (Memmott et al., 2002), and filamentation (yeast-to-hyphal transition) in the pathogenic yeast *Candida albicans* (Dabrowa et al., 1976; Silao et al., 2019).

## Author Contributions

EP and GM contributed to the conception and design of the review. FC, CD’A, AF, OG, and DD performed the literature search, and wrote the first draft of the manuscript. EP and FC prepared the figures. EP and GM critically revised the text and provided substantial scientific contribution. All authors approved the final version of the manuscript.

## Conflict of Interest

The authors declare that the research was conducted in the absence of any commercial or financial relationships that could be construed as a potential conflict of interest.

## Publisher’s Note

All claims expressed in this article are solely those of the authors and do not necessarily represent those of their affiliated organizations, or those of the publisher, the editors and the reviewers. Any product that may be evaluated in this article, or claim that may be made by its manufacturer, is not guaranteed or endorsed by the publisher.

## Abbreviations

AAR: amino acid starvation response
Acetyl-CoA: acetyl coenzyme A
ADCL3: autosomal dominant cutis laxa 3
α-KG: α-ketoglutarate
ALDH18A1: P5C synthase
AMPs: antimicrobial peptides
ARCLII: autosomal recessive cutis laxa type IIB
VitC, ascorbic acid: vitamin C
ATF4: activating transcription factor 4
BDNF: brain-derived neurotrophic factor
BJAB: human lymphoma cell line
CTs: connective tissues
C/EBP: CCAAT/enhancer-binding protein
ChIP-Seq: chromatin immunoprecipitation sequencing
CYR61: cysteine-rich angiogenic inducer 61
DIRICORE: differential ribosome codon reading
DMR: differentially methylated regions
2-DG: 2-deoxy-D-glucose
ECM: extracellular matrix
ECSLC: embryonal carcinoma stem-like cells
EGF: epidermal growth factor
EIF2A: eukaryotic translation initiation factor 2
EIF5A: eukaryotic translation initiation factor 5A
EMT: epithelial-to-mesenchymal transition
ER: endoplasmic reticulum
ERK: extracellular signal-regulated kinase
FAP: fibroblast activation protein
FGF5: fibroblast growth factor 5
FGF8: fibroblast growth factor 8
FGF13: fibroblast growth factor 13
FBS: fetal bovine serum
GABA: γ-aminobutyric acid
GAD: L-glutamate decarboxylase
GCN2: general control non-derepressible 2
GDH: L-glutamate dehydrogenase
GPR142: G-protein -coupled receptor protein 142
HEK293: human embryonic kidney cell line
HeLa: human cervical cancer cell line
HEPG2: human liver cancer cell line
HIF-1 α: hypoxia inducible factor alpha-subunit
HPI and HPII: hyperprolinemia type I and II
IGF-1: insulin-like growth factor 1
ICM: inner cell mass
JMJ: jumonji dioxygenases
Jurkat: human T lymphocyte cell line
mESCs: mouse embryonic stem cells
mTOR: mammalian target of rapamycin
5mC: 5-methyl cytosine
5hmC: 5-hydroxymethyl cytosine
MSCs: mesenchymal stem cells
NEAA: non-essential amino acid
NGF: nerve growth factor
NMDA: *N*-methyl -D-aspartate
NMR: nuclear magnetic resonance
OCD: ornithine cyclodeaminase
PDAC: pancreatic ductal adenocarcinoma
PKDCC: protein kinase domain containing
PHD1-3: HIF prolyl hydroxylases 1-3
P4HA: prolyl-hydroxylase
P5C: Δ 1-pyrroline-5-carboxylate
P5CDH: P5C dehydrogenase
p53: tumor suppressor protein 53
L-Pro: L-proline
PRODH: L-proline dehydrogenase
L-Pro-OH: *trans*-4-hydroxy-L-proline
PRS: prolyl-tRNA synthetase
PYCR: P5C reductase
PREP: prolyl endopeptidase
ROS: reactive oxygen species
SAM: *S*-adenosyl -methionine
SDF-1: stromal cell-derived factor-1
SLC38A2: solute carrier family 38 member 2
SPRR: small proline-rich region protein
TCA: tricarboxylic acid
TET: ten-eleven translocation dioxygenase
TGF β: transforming growth factor beta
UV: ultraviolet
VSMCs: vascular smooth muscle cells
WNT: wingless and/NT-1.
